# Decision tree model to predict one-year survival in ambulatory patients with advanced cancer

**DOI:** 10.1371/journal.pone.0353195

**Published:** 2026-07-14

**Authors:** Yusuke Hiratsuka, Seok-Joon Yoon, Sang-Yeon Suh, Yu Jung Kim

**Affiliations:** 1 Department of Palliative Medicine, Takeda General Hospital, Aizu Wakamatsu, Japan; 2 Department of Palliative Medicine, Tohoku University Graduate School of Medicine, Sendai, Japan; 3 Department of Family Medicine, Chungnam National University Hospital, Daejeon, Republic of Korea; 4 Department of Family Medicine, Dongguk University Ilsan Hospital, Goyang-si, Republic of Korea; 5 Department of Medicine, College of Medicine, Dongguk University, Seoul, Republic of Korea; 6 Division of Hematology and Medical Oncology, Department of Internal Medicine, Seoul National University Bundang Hospital, Seoul National University College of Medicine, Seongnam, Republic of Korea; Karmanos Cancer Institute, Wayne State University School of Medicine, UNITED STATES OF AMERICA

## Abstract

**Background:**

An accurate prognostication is crucial for end-of-life decision-making in advanced cancer care. While existing prognostic tools focus on short-term survival (weeks/months), there is a paucity of studies that have examined the long-term prediction at one year. A one-year timeframe is regarded as a general indicator of palliative care referral; however, there are many uncertain issues. This study aimed to develop a one-year survival prediction model using objective parameters for patients with advanced cancer.

**Methods:**

This was a secondary analysis of data from a Korean prospective cohort study. Participants, with clinician-predicted survival of ≤1 year, were assessed using clinical data, performance status, laboratory data and chemotherapy response. Recursive partitioning analyses (RPA) were used to identify the prognostic factors and build a prediction model.

**Results:**

Of the 200 advanced cancer patients (mean age 64.4, 36% female; 33.5% lung cancer), the median survival was 228 days. Using three variables (chemotherapy response, C reactive protein -Albumin Ratio, and lactate dehydrogenase level), we developed a 4-node survival tree. The model demonstrated an optimism-corrected area under the curve of 0.749 (95% confidence interval: 0.696–0.800) at one year, after 200 bootstrap resampling. The Brier score was 0.161, and the calibration slope was 0.99, indicating high predictive accuracy.

**Conclusions:**

We developed an RPA model to facilitate one-year survival prediction in patients with advanced cancer. The 4-leaf model incorporated only three readily available variables. Following external validation, this model may prove valuable in assisting clinicians with one-year survival prognostication.

## Introduction

Prognostic information holds paramount significance for patients, their families, and healthcare professionals in aiding the end-of-life (EOL) decision-making processes. The life expectancy of a patient can significantly affect their subsequent care strategy, as intricate decisions, such as systemic anticancer therapies and the provision of hospice services, are frequently predicated on prognostic assessments [1–3]. Engaging in prognostic dialogue, underpinned by accurate prognostication, possesses the capacity to enhance shared decision-making and advance care planning initiatives [4–6].

The European Society for Medical Oncology recently disseminated guidelines pertaining to the prognostication of individuals diagnosed with advanced cancer [7]. The modified Glasgow Prognostic Score (mGPS) [8] and Eastern Cooperative Oncology Group Performance Status (ECOG-PS) [9] are recommended for prognostication in patients receiving disease-modifying therapies, with an expected survival of months. However, most previous studies have focused on short-term predictions, such as months or weeks. There is a paucity of studies examining the long-term prediction for one year. A one-year timeframe is regarded as a general indicator of palliative care referral; however, several uncertain issues can arise over a long period [10–12]. Therefore, surprise questions (SQs) have been widely used as screening tool [13]. However, the SQ, a form of clinicians’ prediction of survival is subjective and can thus depend on the clinician’s ability and willingness.

We hypothesized that a one-year survival prediction model based on objective parameters could provide a concrete basis for shared decision-making for patients and families with healthcare professionals. Predicting one-year survival would enable the timely referral of patients to palliative care services, thereby enhancing their quality of life and ensuring the optimal use of medical resources [14]. This study aimed to develop a one-year survival prediction model for patients with advanced cancer.

## Methods

### Participants

This study was a secondary analysis of a prospective cohort study. The objective of this parent study was to develop a prognostic model to facilitate palliative care referral before the last three months of life in outpatients with advanced cancer at medical oncology clinics [15]. Though this study used the same dataset of the parent study, the timeframe of prediction here was one year, thus it was clearly different from that of previous study. The study was conducted at a comprehensive cancer center in Republic of Korea. Patients were selected for convenience; those with advanced cancer treated by medical oncologists at the center were eligible for enrollment during the study period, which spanned from March 2016 to January 2019. The inclusion criteria were as follows: First, a diagnosis of advanced cancer was required. Second, the clinician’s prediction of survival was one year or less. Third, the patient must be an adult (≥18 years). The definition of “advanced cancer” necessitated the presence of recurrent or metastatic disease or progressive locally advanced disease without the possibility of curative treatment. The exclusion criteria were as follows: first, presence of a hematological malignancy was an exclusionary criterion. Second, the clinician’s prediction of survival was limited to four weeks. Third, the patients were deemed incompetent to communicate. The study was conducted in accordance with the Declaration of Helsinki (revised in 2013). Written informed consent was obtained from each patient before enrollment. This study was approved by the Institutional Review Board (IRB) of Seoul National University Bundang Hospital (IRB number: B-1601/332–302).

### Data collection

We obtained demographic data and clinical characteristics, including the primary tumor site and type of chemotherapy, from electronic medical records. After enrollment, the research nurse asked the patients about the presence or absence of dyspnea, dysphagia, anorexia, edema, fatigue, and weight loss. Our survey methods have been previously published elsewhere [15]. The patient performance status was evaluated using the Karnofsky Performance Scale (KPS). Laboratory test results including lactate dehydrogenase (LDH) were collected from the electronic medical records.

The neutrophil-to-lymphocyte ratio (NLR) [16,17] and C-reactive protein (CRP)/albumin ratio (CAR; CRP in mg/dL, albumin in g/dL) [18,19] were calculated as inflammatory and nutritional markers. The body mass index (BMI) was calculated by dividing an individual's weight in kilograms by the square of their height in meters.

The response to chemotherapy was evaluated using the Response Evaluation Criteria in Solid Tumors (RECIST) 1.1 criteria [20]. The RECIST categorizes responses into four distinct categories: partial response (PR), complete response (CR), stable disease (SD), progressive disease (PD) and not evaluable (NE).

Survival time was calculated by subtracting the date of enrollment from the date of death (i.e., death date minus admission date in cases of death or last follow-up date minus admission date in patients alive at follow-up). Patients who were alive at the last follow-up were designated as censored data.

### Statistical analysis

First, we conducted descriptive statistics to summarize the baseline characteristics.

Second, RPA was used to develop a one-year survival tree model [21]. The analysis was tailored to survival data under an exponential distribution. We included all clinically relevant variables for RPA. Those are age, sex, BMI, response of cancer treatment, dyspnea, dysphagia, anorexia, fatigue, edema, weight loss, NLR, LDH, CAR and KPS. Numerical data were dealt as continuous variables. The tree was constructed by recursively splitting the data based on a statistical criterion to optimize the improvement in the log-likelihood function, thereby maximizing the differences in the survival distribution between branches. To prevent overfitting and maintain the balance between model simplicity and predictive accuracy, pruning was performed by adjusting the complexity parameter (cp). The cp value was selected based on the cross-validated relative error to ensure minimal overfitting, while avoiding excessive pruning. Each final node was characterized by an estimated ratio, representing the relative risk of survival of less than one year for the subgroup defined by the node. Higher ratios indicated poorer survival outcomes relative to the all subjects, whereas lower ratios indicated better survival probabilities. Internal validation via 10-fold cross-validation was performed. The code was shared by digital object identifier (https://doi.org/10.5281/zenodo.19281393).

Third, Kaplan-Meier (KM) survival curves were plotted for each final node of the RPA to visualize group-specific survival differences. One year mortality risk for each final node was also calculated.

Finally, model performance at 365 days was evaluated using time-dependent area under the curve (AUC) for discrimination, Brier score for overall accuracy, and calibration analyses. Internal validation was performed using 200 bootstrap resampling to obtain optimism-corrected AUC estimates. Calibration was assessed by comparing predicted and observed 1-year mortality across quintiles of predicted risk using KM estimates, and by estimating the calibration intercept and slope using logistic regression.

All statistical analyses were performed using R (version 4.4.2, R Foundation for Statistical Computing, Vienna, Austria), and statistical significance was set at p < 0.05.

## Results

### Patient demographics

The patient characteristics have been previously reported [15]. In brief, 200 patients were enrolled. The mean age was 64.4 years (range, 32–85 years); 72 patients (36%) were female, and 67 patients (33.5%) were diagnosed with lung cancer. The median time from the diagnosis of advanced cancer to enrollment was 423 days. Within a median follow-up period of 231 days, 159 patients died, and the median overall survival time was 228 days (95% confidence interval [CI]; 199–283 days). The baseline characteristics are summarized in Table 1.

**Table 1:** Characteristics of patients (n = 200).

Variables	n (%) or mean (standard deviation)
Age (years)	64.4 (11.6)
Male	128 (64.0)
Primary cancer site	
Lung	67 (33.5)
Stomach	20 (10.0)
Colon/rectum	28 (14.0)
Breast	18 (9.0)
Ovary/uterine cervix	4 (2.0)
Liver/biliary tract	4 (2.0)
Pancreas	4 (2.0)
Esophagus	5 (2.5)
Head and neck	4 (2.0)
Soft tissue	6 (3.0)
Prostate/bladder/kidney/testis	29 (14.5)
Others	11 (5.5)
Undergoing chemotherapy	131 (65.5)
Response to chemotherapy	
PR	27 (13.5)
SD	72 (36.0)
PD	36 (18.0)
NE	65 (32.5)
Karnofsky Performance Scale	
10-40	0 (0.0)
50	5 (2.5)
60	16 (8.0)
70	68 (34.0)
80	86 (43.0)
90	25 (12.5)
Objective symptoms	
Dyspnea	65 (32.5)
Dysphagia	11 (5.5)
Anorexia	127 (63.5)
Edema	41 (20.5)
Fatigue	148 (74.0)
Weight loss	63 (31.5)
Body mass index (kg/m^2^)	22.40 (3.8)
Laboratory data	
NLR	4.35 (4.7)
CAR	0.77 (1.3)
LDH (IU/L)	327.3 (395.4)
Survival time (days)	228 (199.0–283.0) ^a)^

PR, partial response; SD, stable disease; PD, progressive disease; NE, not evaluable; NLR, neutrophil-to-lymphocyte ratio; CAR, serum C-reactive protein (mg/dL) to serum albumin (g/dL) ratio; LDH, serum lactate dehydrogenase level.

^a)^Data are expressed as medians (95% confidence intervals).

### 4-leaf RPA model

**Fig 1:**
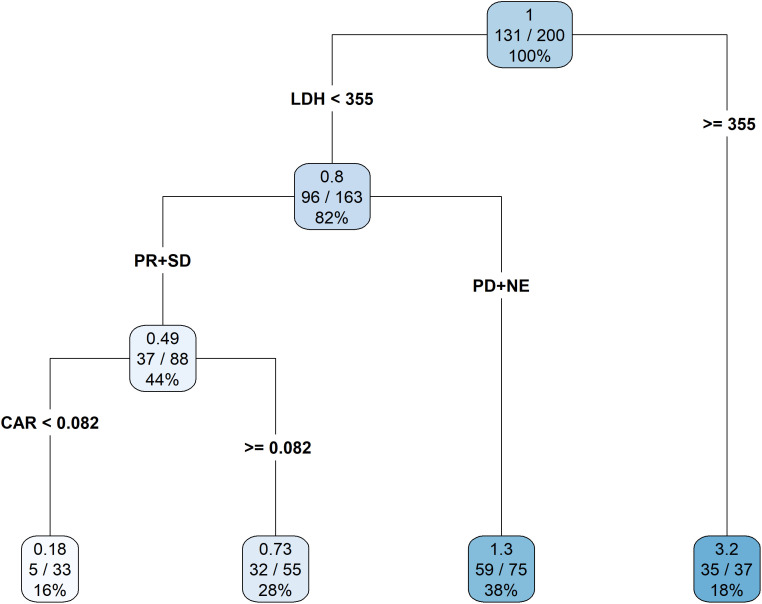
Recursive partitioning analysis. PR, partial response; SD, stable disease; PD, progression of disease; NE, not evaluable.

Fig 1 shows the survival tree from the RPA. Three variables (response to chemotherapy, CAR and LDH) were selected from the RPA. Finally, the tree had 4 final leaves (nodes). Each node was defined as meeting following conditions. Final node 1 was assigned if LDH < 355 & response to chemotherapy = PR + SD & CAR < 0.082. Final node 2 required LDH < 355 & response to chemotherapy = PR + SD & CAR >=0.082. Final node 3 was defined as LDH < 355 & response to chemotherapy = PD + NE. Final node 4 was assigned if LDH >=355. The estimated ratios for each node were 0.18, 0.73, 1.3, and 3.2, respectively. The baseline for these ratios was the overall event (survival less than 1 year) in the total cohort (131 events in 200 patients). These ratios ranged from 0.18 to 3.2, reflecting substantial heterogeneity in survival outcomes across the subgroups

Numbers in the first line of the nodes are estimated rates representing the relative risk of survival of less than one year for the subgroup divided by the node. The baseline for these ratios was the overall event rate for the entire cohort (131 events in 200 patients). Higher ratios indicated poorer survival outcomes relative to the baseline, whereas lower ratios indicated better survival probabilities. The numbers middle in the nodes are expressed as events/observations and the numbers lowest in the nodes represents proportion of patients (events/observations).

### Survival difference among the final leaves from the survival tree

**Fig 2:**
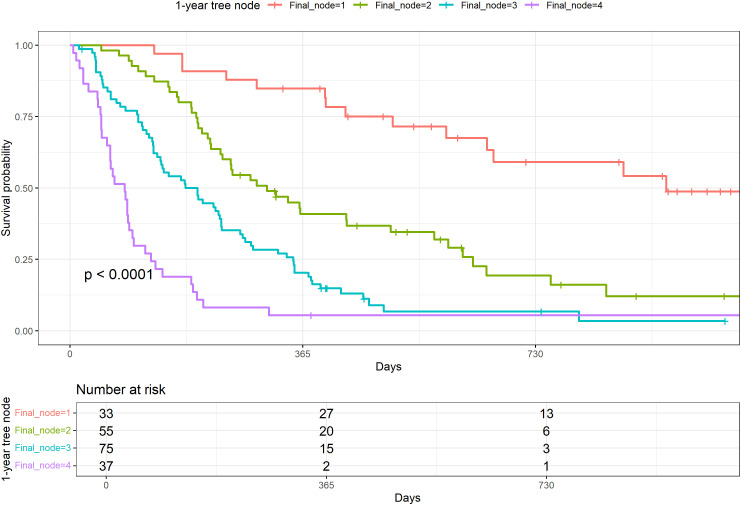
Kaplan-Meier survival curves of the final nodes from recursive partitioning analysis for survival less than one year.

Fig 2 shows the survival curves of each final node for a survival period of less than one year. Survival differences among final nodes were confirmed using the log-rank test (p < 0.001). The final RPA model resulted in 4 final nodes (Fig 1). The estimated 1-year mortality risk for each node was as follows. For final node 1, the risk was 15.15 (95% confidence interval (CI):1.99–26.54)%. Final node 2 showed risk of 59.18 (95% CI: 43.56–70.47)%. Final node 3 had risk of 79.73 (95% CI: 68.15–87.10) %, and that of final node 4 was 94.59 (95% CI: 79.19–98.60) %.

Nodes 1–4 indicate the final nodes from the recursive partitioning analysis (from left to right in Fig 1). The median survival times for node 1,2,3 and node 4 were 935 (95% confidence interval (CI): 654-not applicable), 309 (239–571) days, 200 (142–237) days, and 86 (63–100) days, respectively. The estimated 1-year mortality risk for node 1,2,3 and node 4 was: 15.15 (95% CI: 1.99–26.54)%, 59.18 (95% CI: 43.56–70.47)%, 79.73 (95% CI: 68.15–87.10)%, and 94.59 (79.19–98.60)%, respectively. The p-value was < 0.001 from the log-rank test.

### Performance and calibration

**Fig 3:**
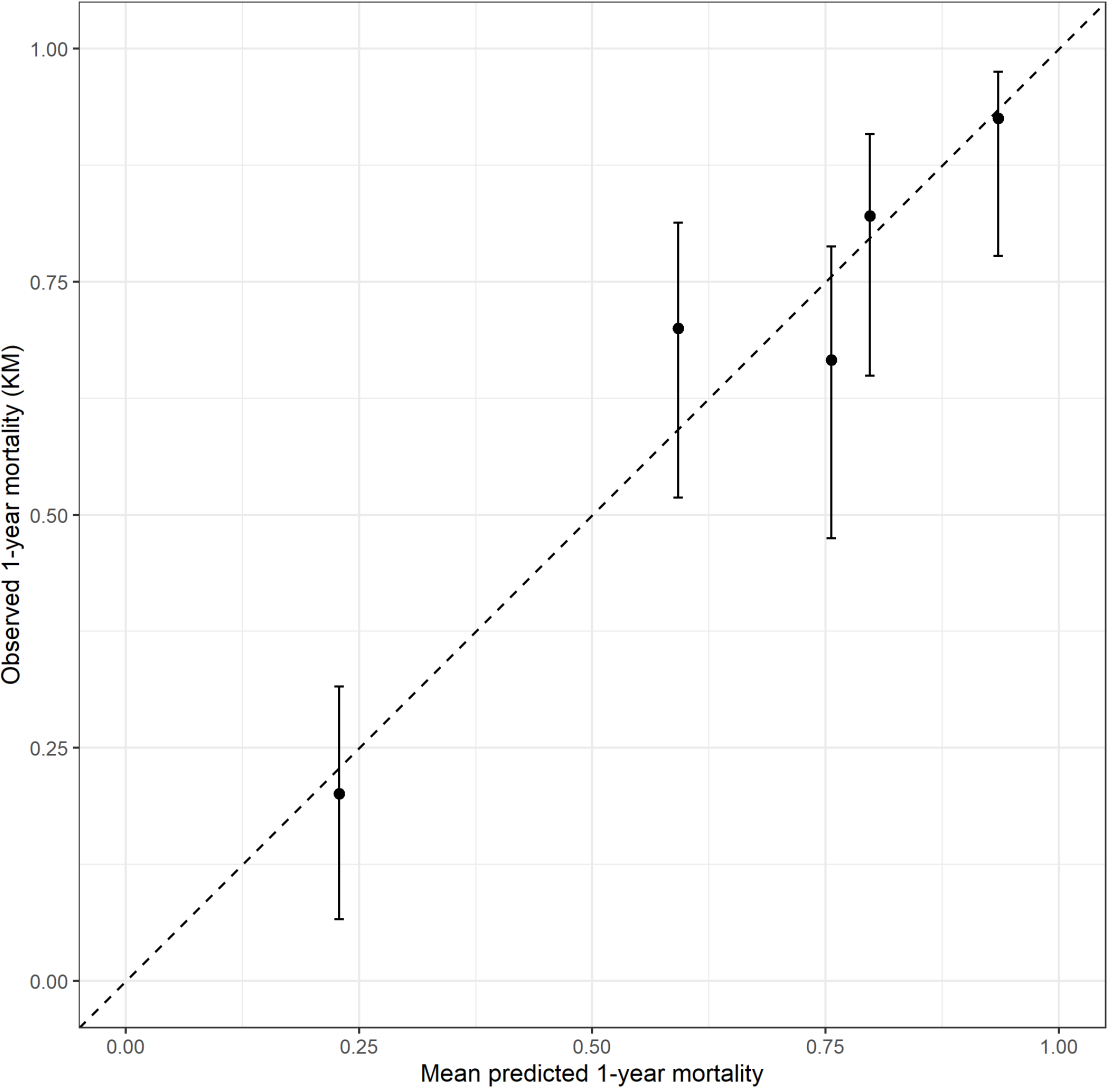
Calibration plot at 1 year.

To evaluate model performance, optimism corrected time-dependent AUC and Brier score were calculated at one year, yielding 0.749 (95% CI 0.696–0.800, mean optimism was 0.047) and 0.161, respectively. Furthermore, the calibration plot showed excellent agreement between predicted and observed mortality, with a slope of 0.99 and an intercept of −0.04 (Fig 3).

Patients were grouped into quintiles according to predicted 1-year mortality risk. Points represent the mean predicted probability within each group, and observed probabilities were estimated using the Kaplan–Meier method. Error bars indicate 95% confidence intervals. The dashed line represents perfect calibration. The calibration slope was 0.99 and the intercept was −0.04.

## Discussion

We successfully developed a novel decision tree model to predict one-year survival in outpatients with advanced cancer. It is simple and practical that four easily assessed clinical data (response to chemotherapy, LDH and CAR) can guide the prediction of one-year survival.

Our components are essentially checked in clinical practice, one history (chemotherapy response), and two laboratory values (CAR, LDH). For instance, if a patient has LDH < 355, a chemotherapy response of PR or SD, and CAR < 0.08, then the probability of one-year mortality is reduced to 18% of the probability compared to that of all participants. Conversely, if a patient has LDH ≥ 355, the probability of dying within a year increases to 320% compared to the average probability of all participants.

These three variables are consistent with those of previous studies in terms of poor prognosis in patients with advanced cancer. LDH is a valuable prognostic tool for different types of cancer, especially those in advanced stages [18,19,22]. Its discriminatory ability has been reported to be comparable to that of the Glasgow Prognostic Score, which is also known as the simple inflammation-based score [22]. The RECIST criteria have been widely used to assess the treatment response in patients with cancer; however, their association with prognosis has been investigated in several studies [23–25]. Elevated LDH levels are consistently associated with poor overall survival and progression-free survival in several types of advanced cancers [26,27]. LDH levels have also been found to be significantly associated with performance status, systemic inflammation, and survival in patients with advanced cancer [28].

A clinical prediction model for one-year survival in patients with advanced cancer was recently developed [11]. The model provides a nomogram with 12 variables, including a surprise question. Compared with the nomogram, our decision tree consists of fewer and more objective items. Thus, the decision tree provides an easy-to-use interpretable model [28], with advantages including being intuitive and easily understood by clinicians; therefore, it can facilitate prognostic communication and advance care planning.

Our study had some limitations. First, it was conducted at a single tertiary cancer center with a heterogeneous group of patients in Republic of Korea. Thus, the study design may limit generalizability. For routine clinical decision-making, the decision tree should be viewed as an internally validated prognostic tool that requires external validation in independent cohorts later. Second, patients were enrolled based on the physicians’ prediction of one-year survival. Thus, participant selection bias might have occurred and influenced the observed associations because of overestimation, although the current study aimed to develop a one-year prediction model. Third, patients in low-risk nodes could survive more than a few years despite inclusion criteria; less than one year’s survival. Unexpectedly good responses to new chemotherapy agents, such as immunotherapy or targeted therapy, may have contributed to the prolonged survival. Last, many years have passed since the initial patient enrollment and the reporting of the study results. We acknowledge that our results may not fully reflect the potential impact of innovative treatments such as immunotherapy and currently available antibody-drug conjugates.

## Conclusion

We developed an RPA model to facilitate the one-year survival prediction in patients with advanced cancer. The 4-leaf model included only three simple variables: LDH level, response to chemotherapy, and CAR. With further external validation, the model may be useful for assisting clinicians in prediction of one-year survival.

## Abbreviations

EOL: end-of-life
mGPS: modified Glasgow Prognostic Score
ECOG-PS: Eastern Cooperative Oncology Group Performance Status
SQ: surprise question
IRB: Institutional Review Board
KPS: Karnofsky Performance Scale
NLR: neutrophil-to-lymphocyte ratio
CRP: C-reactive protein
CAR: C-reactive protein /albumin ratio
BMI: body mass index
RECIST: Response Evaluation Criteria in Solid Tumors
PR: partial response
CR: complete response
SD: stable disease
PD: progressive disease
NE: not evaluable
LDH: lactate dehydrogenase
RPA: recursive partitioning analysis
cp: complexity parameter
CI: confidence interval
AUC: area under the curve
