# Supplementary material for: Decision tree model to predict one-year survival in ambulatory patients with advanced cancer
Source: PLoS One. 2026 Jul 14;21(7):e0353195. doi: 10.1371/journal.pone.0353195 (PMC13367727; doi:10.1371/journal.pone.0353195)
Supplement: Attachment — Submitted filename: Response to Reviewers.docx [file pone.0353195.s002.docx]

Thank you for the opportunity to revise our manuscript. We have carefully addressed the comments of the reviewers and listed our point-by-point responses to each below. We express our sincere appreciation for your time and effort and providing your insightful guidance. Our responses are shown in blue.

Response to the Journal Requirements:

Requirement 1:

Please ensure that your manuscript meets PLOS ONE's style requirements, including those for file naming.

Response 1:

Thank you for your comments. We have modified the manuscript accordingly.

Requirement 2:

Please include your tables as part of your main manuscript and remove the individual files. Please note that supplementary tables (should remain/ be uploaded) as separate "supporting information" files.

Response 2:

Thank you for your advice. We’ve included our table(Table 1) as part of our main manuscript and removed the individual files.

Requirement 3:

Please note that PLOS One has specific guidelines on code sharing for submissions in which author-generated code underpins the findings in the manuscript. In these cases, we expect all author-generated code to be made available without restrictions upon publication of the work. Please review our guidelines at https://journals.plos.org/plosone/s/materials-and-software-sharing#loc-sharing-code and ensure that your code is shared in a way that follows best practice and facilitates reproducibility and reuse.

Response 3:

Thank you very much for your advice. We shared our code accordingly and inserted DOI in the manuscript (<https://doi.org/10.5281/zenodo.19281393>) (p.7, lines 140–141).

Requirement 4:

Your ethics statement should only appear in the Methods section of your manuscript. If your ethics statement is written in any section besides the Methods, please delete it from any other section.

Response 4:

Thank you for your comment. We’ve removed the ethics statement in Declaration section accordingly.

Requirement 5:

We noted in your submission details that a portion of your manuscript may have been presented or published elsewhere. Yes, total dataset was used to publicate a related manuscript. However, the aim was to develop 3-month prediction of survival. This work is different in terms of time-frame. We aimed to develop 1-year survival prediction based on the same dataset. Please clarify whether this [conference proceeding or publication] was peer-reviewed and formally published. If this work was previously peer-reviewed and published, in the cover letter please provide the reason that this work does not constitute dual publication and should be included in the current manuscript.

Response 5:

We appreciate your concern. As you pointed out, we have published the relevant article that aimed to develop 3-month prediction of survival (https://doi.org/10.4143/crt.2021.483). It is peer-reviewed and formally published. We clarified this in current manuscript (p,5, lines 83–87) and the cover letter, too. Though this work used the same dataset, it aimed to develop 1-year survival prediction model. This time-frame is different and predicting model is a brand new one. Therefore, this study had a different research aim, different analysis and results. Thus this does not constitute dual publication.

Requirement 6:

We note that you have indicated that there are restrictions to data sharing for this study. For studies involving human research participant data or other sensitive data, we encourage authors to share de-identified or anonymized data. However, when data cannot be publicly shared for ethical reasons, we allow authors to make their data sets available upon request. Please update your Data Availability statement in the submission form accordingly.

a) If there are ethical or legal restrictions on sharing a de-identified data set, please explain them in detail (e.g., data contain potentially identifying or sensitive patient information, data are owned by a third-party organization, etc.) and who has imposed them (e.g., a Research Ethics Committee or Institutional Review Board, etc.). Please also provide contact information for a data access committee, ethics committee, or other institutional body to which data requests may be sent.

b) If there are no restrictions, please upload the minimal anonymized data set necessary to replicate your study findings to a stable, public repository and provide us with the relevant URLs, DOIs, or accession numbers

Response 6:

Thank you very much for your advice. The local Institutional Review Board (IRB) prohibited to share patient data publicly, because it contains sensitive patient information (survival status, treatment history so on). Original written consent forms from patients didn’t include to share data with others not related to this study directly. Thus it is violation of protection of privacy, according to the local IRB, Seoul National University Bundang Hospital Institutional Review Board. We only can provide the data upon request, and added the contact information of IRB.

The contact information of IRB is as below:

Seoul National University Bundang Hospital Institutional Review Board

Telephone: 082-031-787-8801

Address: 82, Gumi-ro 173 beon-gil, Bundang-gu, Seongnam-si, Gyeonggi-do, 13620, Republic of Korea

Requirement 7:

In the online submission form, you indicated that the datasets used and/or analyzed during the current study are available from the corresponding author on reasonable request. All PLOS journals now require all data underlying the findings described in their manuscript to be freely available to other researchers, either 1. In a public repository, 2. Within the manuscript itself, or 3. Uploaded as supplementary information. This policy applies to all data except where public deposition would breach compliance with the protocol approved by your research ethics board. If your data cannot be made publicly available for ethical or legal reasons (e.g., public availability would compromise patient privacy), please explain your reasons on resubmission and your exemption request will be escalated for approval.

Response 7:

Thank you very much for your advice. As we stated above response 6, public reposition breaches compliance with the protocol approved by our research ethics board. According to the law of privacy protection in Korea, we clarified that patient data would be accessed by only authorized personnel directly related to the study. The law of privacy protection in Korea is quite strict, and it has been reinforced recently. Moreover, the dataset contained sensitive information such as treatment responses, survival status etc. Our research ethics board required that those statements were included in written consent form for patients. Therefore, public availability of this dataset is neither compatible with ethics, nor legal reasons in Korea. We seek your generous understanding about this matter.

Requirement 8:

We note that the grant information you provided in the ‘Funding Information’ and ‘Financial Disclosure’ sections do not match. When you resubmit, please ensure that you provide the correct grant numbers for the awards you received for your study in the ‘Funding Information’ section.

Response 8:

Thank you for pointing this out. We apologize for the mistake and have confirmed the relevant description (p.15, lines 297–299).

Requirement 9:

We note that there is identifying data in the Supporting Information file <2021_CRT_Px_Model_EPC.pdf>. Due to the inclusion of these potentially identifying data, we have removed this file from your file inventory. Prior to sharing human research participant data, authors should consult with an ethics committee to ensure data are shared in accordance with participant consent and all applicable local laws. Data sharing should never compromise participant privacy. It is therefore not appropriate to publicly share personally identifiable data on human research participants. Please remove or anonymize all personal information (<specific identifying information in file to be removed>), ensure that the data shared are in accordance with participant consent, and re-upload a fully anonymized data set. Please note that spreadsheet columns with personal information must be removed and not hidden as all hidden columns will appear in the published file.

Response 9:

Thank you for your opinion. However, the file is an accepted manuscript of a published article, it is not a dataset. It does not contain any personal information. There are only tables and figures published, no columns for identifying data. We changed the file as an officially published article as Supporting Information, to avoid possible misunderstanding.

Requirement 10:

Please include captions for your Supporting Information files at the end of your manuscript, and update any in-text citations to match accordingly.

Response 10:

Thank you for your comment. As we stated above Response 9, Supporting Information file was an accepted manuscript of previously published article using the same dataset. We replaced the file as the officially published file. We included captions for our Supporting Information file at the end of our manuscript. We mentioned the reference as 15 in the reference list.

Requirement 11:

If the reviewer comments include a recommendation to cite specific previously published works, please review and evaluate these publications to determine whether they are relevant and should be cited. There is no requirement to cite these works unless the editor has indicated otherwise.

Response 11:

Thank you for your advice. We understand your requirement. No reviewers recommended more citations.

Response to the reviewer’s comments:

**Reviewer 1**

Comment 1:

The manuscript restricts the variables entered into the RPA to only those that were statistically significant in the multivariable Cox regression model. This approach is not appropriate for tree based modeling. RPA is designed to detect nonlinear effects, threshold based relationships, and higher order interactions that may not appear significant in a Cox model, which assumes log linearity and does not automatically evaluate interactions. Limiting RPA inputs to only Cox significant variables risks excluding clinically important predictors, introduces bias through data driven preselection, and may reduce the stability and interpretability of the resulting tree.

Response 1:

We appreciate you pointing this out. According to your advice, we applied a new analysis method. First, we put all clinically possible variables – demographic variables, previous treatment effect, symptoms and signs, performance status laboratory tests -- in RPA (p.6, line 126–p.7, line 130). The new results are shown in Abstract (p.2, lines 41–42), Results (p.10, lines 169–179) and Figure 1,2.

Comment 2:

The same dataset is used for both (1) selecting prognostic factors via Cox regression and (2) developing the RPA prediction model. This constitutes a form of double dipping, in which the data are used twice for variable selection and model construction. Such reuse increases the risk of overfitting, inflates the apparent importance of selected variables, and undermines the validity of the final RPA model. Prognostic factor identification and predictive model development should be conceptually and analytically separated, or at minimum, variable selection should not be based solely on significance testing within the same dataset.

Response 2:

Thank you very much for your advice. According to your advice, we decided to change analysis method and used all possible variables in RPA (p.6, line 126–p.7, line 130). The new results are shown in Figure 1 and 2.

Comment 3:

A more robust strategy would be to include all clinically relevant variables (or all variables with acceptable completeness and variability) in the RPA and allow the algorithm to perform its own variable selection. The authors should revise the modeling strategy accordingly and provide justification for the final set of variables included in the tree.

Response 3:

Thank you for your valuable advice. According to your advice, we used all clinically relevant variables (demographic variable, treatment response, symptoms and signs, performance status, body mass index, and laboratory tests) in the RPA (p.6, line 126–p.7, line 130). Those variables were all collected data we had indeed, except tumor type. The new results are shown in Figure 1 and 2.

**Reviewer 2**

Comment 1:

The Data Availability Statement says: “available from corresponding author on reasonable request,” which is generally not sufficient under PLOS data policy unless justified.

Response 1:

Thank you very much for your advice. The public reposition breaches compliance with the protocol approved by our research ethics board. We clarified that patient data would be accessed by only authorized personnel directly related to the study in the protocol. The law of privacy protection in Korea is quite strict, it has been reinforced recently and it rules the research ethics board. Moreover, the dataset contained sensitive information such as treatment responses, and survival status etc. Our research ethics board required that those statements about privacy protection were included in written consent form for patients. Therefore, public availability of this dataset is neither compatible with ethics, nor legal restrictions in Korea. We seek your generous understanding about this matter.

Comment 2:

While the decision tree is intuitive, providing more granular performance metrics would make it more useful for clinicians. Reporting Sensitivity and Specificity.

Response 2:

We appreciate your advice. For calculating sensitivity and specificity, we need 2*2 table, however the final nodes are 4 nodes. Thus, we need to make the final node to binary variables; i.e. [final node 4] vs. [other nodes], [final node 3 and 4] vs. [node 1 and 2], [final node 2, 3, and 4] vs. [node 1]. Results are as below.

| Predictive value | Sensitivity | Specificity | PPV | NPV | Accuracy |
| --- | --- | --- | --- | --- | --- |
| Only 4 | 0.267 | 0.971 | 0.946 | 0.411 | 0.510 |
| 3 and 4 | 0.718 | 0.739 | 0.839 | 0.580 | 0.725 |
| 2, 3, and 4 | 0.962 | 0.406 | 0.754 | 0.848 | 0.770 |

We are afraid that these binary classifications do not fit our research aim and analysis model. We do not think these results are appropriate to interpret our analysis. Because we’re interested in each node, grouping nodes seems arbitrary to us. It would be better to show risk of each node calculated in KM curve. Thus, we have decided not to show the results. We hope your generous understanding.

Comment 3:

The authors acknowledge that the study was conducted at a single tertiary center in South Korea. To make the model globally relevant, it should be tested on an independent dataset from a different geographic region or clinical setting. If an external dataset is unavailable, the authors should perform internal validation techniques like k-fold cross-validation or bootstrapping.

Response 3:

Thank you very much for your valuable advice. We performed 10-fold cross-validation (p.7, lines 139–140) and have added the calibration plot as Figure 3.

Comment 4:

Some places say CRP <0.08 instead of CAR <0.08 when describing node definitions. Please check carefully for consistent variable naming.

Response 4:

Thank you for pointing this out. We apologize for the inconsistency. We corrected them accordingly.

Comment 5:

Provide model details for reproducibility;

o the R package used for RPA (e.g., rpart, partykit, etc.),

o the selected complexity parameter (cp) value,

o minimum node size, splitting criteria,

o and code or pseudo-code if possible.

Response 5:

Thank you for your suggestion. We’ve added the details in the code and provided it as DOI (<https://doi.org/10.5281/zenodo.19281393>) in the manuscript (p.7, lines 140–141).

Comment 6:

Figure 1 and Figure 2 appear low resolution and hard to read (node text, axis labels, legend). Please improve figure quality for publication (higher DPI, larger fonts, consistent color/line styles).

Response 6:

Thank you for your recommendation. We’ve improved the figure quality accordingly and resubmitted those.

Comment 7:

Another problem is that the Data availability statement may not be sufficient for PLOS data policy. With minor revisions focused on clarifying model details, adding model validation metrics, and improving figures, the manuscript would be stronger and more reproducible.

Response 7:

Thank you very much for your advice. As we stated in Response 1, the public reposition breaches compliance with the protocol approved by our research ethics board. We clarified that patient data would be accessed by only authorized personnel directly related to the study in our protocol. The law of privacy protection in Korea is quite strict, and it has been reinforced recently. Moreover, the dataset contained sensitive information such as treatment responses, survival status etc. Our research ethics board required that those statements were included in written consent form for patients. Therefore, public availability of this dataset is neither compatible with ethics, nor legal reasons in Korea. We seek your generous understanding about this matter.

**Reviewer 3**

Comment 1:

Several limitations should be more clearly emphasized when interpreting the results.

1) This is a secondary analysis of a single-center cohort with a relatively modest sample size and heterogeneous cancer types, which may limit generalizability.

2) Inclusion based on clinician-predicted survival of ≤1 year introduces potential selection bias, as clinician judgment is subjective and may influence the observed associations.

3) Although pruning and cross-validation were performed, the decision tree model remains at risk of overfitting, particularly given the data-driven selection of cut points for continuous variables. The absence of internal optimism correction and external validation further limits conclusions regarding predictive performance and clinical utility.

Response 1:

We appreciate your suggestion. Regarding 1) and 2), we have added some explanation in the section of limitation (p 13, lines 246-247 and p. 13, lines 249-250). As for 3), we have added time-dependent AUC of the decision tree, Brier score, and calibration (p.7, lines 145–150 and p.11, line 208– p 12, line 211).

Comment 2:

The manuscript reports an overall median survival of 7.5 months, while Figure 2 presents median survival times for some terminal nodes exceeding 900 days. Although this may reflect heterogeneity across risk strata, the use of different time units (months vs days) is potentially confusing. The authors should standardize The time units across the manuscript and clarify how long survival in low-risk nodes aligns with the inclusion criterion of clinician-predicted survival ≤1 year.

Response 2:

Thank you for your advice. We have united the time units as days (p.8, lines 159–161). We added possible explanation for the long survival in low-risk nodes in the manuscript (p.13, lines 251–254).

Comment 3:

The Data Availability Statement indicates that data are available upon reasonable request, which does not meet PLOS ONE data-sharing requirements. If participant privacy restricts full public sharing, the authors should explicitly state these limitations and consider providing a de-identified dataset or depositing the data in a controlled-access repository.

Response 3:

Thank you very much for your advice. The public reposition breaches compliance with the protocol approved by our research ethics board. We clarified that patient data would be accessed by only authorized personnel directly related to the study in our protocol. The law of privacy protection in Korea is quite strict, it has been reinforced recently, and it rules research ethics. Moreover, the dataset contained sensitive information such as treatment responses, survival status etc. Our research ethics board required that those statements were included in written consent form for patients. Therefore, public availability of this dataset is neither compatible with ethics, nor legal reasons in Korea. We seek your generous understanding about this matter.

Comment 4:

The manuscript is generally well written and clearly organized, with only (1) minor grammatical or stylistic issues that can be addressed during revision (e.g., "LDH levels are also been found..." --> This should be corrected to "LDH levels have also been found to be..."). Figures and tables are clear and appropriately support the text. Some other consistency minor issues need to be fixed as well: (2 Variable Terminology: On page 9, the text describes "Node 2" as having "CRP < 0.08". However, the study focuses on CAR (C-reactive protein/albumin ratio), not raw CRP. This should be corrected for consistency to avoid confusing the two laboratory measures.; (3) Abbreviations: While a list is provided, ensuring that all abbreviations (like CAR and RPA) are consistently defined at their first mention in both the abstract and main text would improve readability.

Response 4:

We appreciate your valuable advice. Thank you for your encouragement. We have corrected our mistakes as you pointed out. As for abbreviations, we have checked for readability.

Closing comments to the editor：

We appreciate the opportunity to revise our manuscript for publication in *PLOS ONE* and hope that its revised version meets the journal's standards.
