# Supplementary material for: Decision tree model to predict one-year survival in ambulatory patients with advanced cancer
Source: PLoS One. 2026 Jul 14;21(7):e0353195. doi: 10.1371/journal.pone.0353195 (PMC13367727; doi:10.1371/journal.pone.0353195)
Supplement: Attachment — Submitted filename: Response letter_R2(3).docx [file pone.0353195.s003.docx]

Thank you for the opportunity to revise our manuscript. We have carefully addressed the comments of the reviewers and listed our point-by-point responses to each below. We express our sincere appreciation for your time and effort and providing your insightful guidance. Our responses are shown in blue.

Response to the Journal Requirements:

Requirement 1:

If the reviewer comments include a recommendation to cite specific previously published works, please review and evaluate these publications to determine whether they are relevant and should be cited. There is no requirement to cite these works unless the editor has indicated otherwise.

Response 1:

Thank you for your comment. There were no recommendations regarding references from the two reviewers.

Requirement 2:

Please review your reference list to ensure that it is complete and correct. If you have cited papers that have been retracted, please include the rationale for doing so in the manuscript text, or remove these references and replace them with relevant current references. Any changes to the reference list should be mentioned in the rebuttal letter that accompanies your revised manuscript. If you need to cite a retracted article, indicate the article’s retracted status in the References list and also include a citation and full reference for the retraction notice.

Response 2:

We thank your advice. We have confirmed reference list accordingly.

Requirement 3:

Regarding the Data Availability (mentioned by both reviewers), the authors explained in their responses but did not provide a detailed explanation in the Data Availability Statement. The authors need to update the Data Availability Statement following PLOS One policy: https://journals.plos.org/plosone/s/data-availability.

"If there are ethical or legal restrictions on sharing a sensitive data set, authors should provide the following information within their Data Availability Statement upon submission:

- Explain the restrictions in detail (e.g., data contain potentially identifying or sensitive patient information)

- Provide contact information for a data access committee, ethics committee, or other institutional body to which data requests may be sent".

Response 3:

We appreciate you pointing this out. We have added the explanation in the section of “Data availability statement” (p.15-16, lines 307–317).

Requirement 4:

The two reviewers mentioned a couple of remaining issues, which I hope the authors can address.

Response 4:

Thank you for your comments. We have addressed the issues accordingly.

Response to the reviewer’s comments:

**Reviewer #2**

Comment 1:

First, although the authors provided a detailed explanation regarding the restrictions on public data sharing due to IRB requirements, patient consent limitations, and Korean privacy regulations, I would like the editor to determine whether the revised Data Availability Statement sufficiently satisfies the journal’s data sharing policy requirements.

Response 1:

Thank you for your advice. We have revised the “Data availability statement” accordingly (p.15-16, lines 307–317).

Comment 2:

Second, while the figures are now generally readable and improved compared with the original submission, the resolution and overall image quality still appear somewhat suboptimal for publication. I would appreciate the editor’s assessment on whether the current figure quality is acceptable for publication standards or if higher-resolution versions should still be requested.

Response 2:

We thank your valuable advice. We have submitted high-resolution versions of figures as possible.

**Reviewer #3**

Comment 1:

n my earlier review I emphasized several limitations that needed clearer discussion: the secondary analysis of a single‑center cohort with a modest sample size and heterogeneous cancer types, the potential selection bias from enrollment based on clinician‑predicted survival ≤1 year, and the risk of overfitting in a data‑driven decision tree model without optimism correction or external validation. In the revision, you have expanded the limitations section to discuss these points more explicitly, and you now present time‑dependent AUC, Brier score, and calibration metrics with internal validation. This is a substantial improvement and better frames the strength and boundaries of the conclusions.

The standardization of time units to days throughout the manuscript and the clarification of why some low‑risk nodes show much longer survival than the overall median directly addresses my previous concern about potential confusion between months and days. Your added explanation about possible reasons for longer survival in the low‑risk group (e.g., treatment responses) is reasonable and appropriately cautious.

I also note improvements in clarity and language. The earlier example I mentioned (“LDH levels are also been found…”) and the inconsistency between CRP and CAR in node descriptions have been corrected, and abbreviations appear to be defined more consistently. The overall structure and flow are clear, and the figures are now more legible.

Response 1:

Thank you very much for your comments. We would like to express our gratitude for your previous valuable comments.

Comment 2:

One point that still merits attention is how you frame the clinical applicability of the model. Even with the improved internal validation, this remains an internally validated model derived from a single institution. I recommend that you slightly soften the language in the Discussion and Conclusions to state explicitly that the decision tree should be viewed as an internally validated prognostic tool that requires external validation in independent cohorts before it can be used routinely in clinical decision‑making.

Response 2:

We appreciate your advice. We totally agree with you. We have added the explanation in the section of limitation (p 14 lines 263-264)

Comment 3:

Finally, I acknowledge your explanation regarding the constraints on data sharing imposed by local privacy law and your IRB. While the ultimate acceptability of this arrangement lies with the journal, it would be helpful if the Data Availability Statement in the manuscript itself clearly and succinctly states: (i) that public deposition of the dataset is not permitted by the IRB; (ii) that any data access must occur under IRB or institutional oversight; and (iii) how interested researchers should initiate such a request. Making this information as specific and transparent as possible will support readers’ understanding of the limitations around data availability.

Response 3:

Thank you for your advice. We have revised the “Data availability statement” accordingly (p15-16, lines 307-317).

Closing comments to the editor：

We appreciate the opportunity to revise our manuscript for publication in *PLOS One* and hope that its revised version meets the journal's standards.
