# Supplementary material for: Decision tree model to predict one-year survival in ambulatory patients with advanced cancer
Source: PLoS One. 2026 Jul 14;21(7):e0353195. doi: 10.1371/journal.pone.0353195 (PMC13367727; doi:10.1371/journal.pone.0353195)
Supplement: Peer review history

# Peer review: Decision Letter 0

**DOI:** 10.1371/journal.pone.0353195.r001

**Authors:** Yang Shi

16 Feb 2026

PONE-D-25-58729

Decision Tree Model to Predict One-Year Survival in Ambulatory Patients with Advanced Cancer

PLOS One

Dear Dr. Suh,

Thank you for submitting your manuscript to PLOS ONE. After careful consideration, we feel that it has merit but does not fully meet PLOS ONE’s publication criteria as it currently stands. Therefore, we invite you to submit a revised version of the manuscript that addresses the points raised during the review process.

Please submit your revised manuscript by Apr 01 2026 11:59PM. If you will need more time than this to complete your revisions, please reply to this message or contact the journal office at plosone@plos.org. When you're ready to submit your revision, log on to https://www.editorialmanager.com/pone/ and select the 'Submissions Needing Revision' folder to locate your manuscript file.

Please include the following items when submitting your revised manuscript:

- A letter that responds to each point raised by the academic editor and reviewer(s). You should upload this letter as a separate file labeled 'Response to Reviewers'.
- A marked-up copy of your manuscript that highlights changes made to the original version. You should upload this as a separate file labeled 'Revised Manuscript with Track Changes'.
- An unmarked version of your revised paper without tracked changes. You should upload this as a separate file labeled 'Manuscript'.

If you would like to make changes to your financial disclosure, please include your updated statement in your cover letter. Guidelines for resubmitting your figure files are available below the reviewer comments at the end of this letter.

If applicable, we recommend that you deposit your laboratory protocols in protocols.io to enhance the reproducibility of your results. Protocols.io assigns your protocol its own identifier (DOI) so that it can be cited independently in the future. For instructions see: https://journals.plos.org/plosone/s/submission-guidelines#loc-laboratory-protocols. Additionally, PLOS ONE offers an option for publishing peer-reviewed Lab Protocol articles, which describe protocols hosted on protocols.io. Read more information on sharing protocols at https://plos.org/protocols?utm_medium=editorial-email&utm_source=authorletters&utm_campaign=protocols.

We look forward to receiving your revised manuscript.

Kind regards,

Yang Shi, PhD

Academic Editor

PLOS One

Journal Requirements:

When submitting your revision, we need you to address these additional requirements.

1. Please ensure that your manuscript meets PLOS ONE's style requirements, including those for file naming. The PLOS ONE style templates can be found at

https://journals.plos.org/plosone/s/file?id=wjVg/PLOSOne_formatting_sample_main_body.pdf and https://journals.plos.org/plosone/s/file?id=ba62/PLOSOne_formatting_sample_title_authors_affiliations.pdf

2. Please include your tables as part of your main manuscript and remove the individual files. Please note that supplementary tables (should remain/ be uploaded) as separate "supporting information" files.

3. Please note that PLOS One has specific guidelines on code sharing for submissions in which author-generated code underpins the findings in the manuscript. In these cases, we expect all author-generated code to be made available without restrictions upon publication of the work. Please review our guidelines at https://journals.plos.org/plosone/s/materials-and-software-sharing#loc-sharing-code and ensure that your code is shared in a way that follows best practice and facilitates reproducibility and reuse.

4. Your ethics statement should only appear in the Methods section of your manuscript. If your ethics statement is written in any section besides the Methods, please delete it from any other section.

5. We noted in your submission details that a portion of your manuscript may have been presented or published elsewhere. Yes, total dataset was used to publicate a related manuscript. However, the aim was to develop 3-month prediction of survival. This work is different in terms of time-frame. We aimed to develop 1-year survival prediction based on the same dataset.

Please clarify whether this [conference proceeding or publication] was peer-reviewed and formally published. If this work was previously peer-reviewed and published, in the cover letter please provide the reason that this work does not constitute dual publication and should be included in the current manuscript.

6. We note that you have indicated that there are restrictions to data sharing for this study. For studies involving human research participant data or other sensitive data, we encourage authors to share de-identified or anonymized data. However, when data cannot be publicly shared for ethical reasons, we allow authors to make their data sets available upon request. For information on unacceptable data access restrictions, please see http://journals.plos.org/plosone/s/data-availability#loc-unacceptable-data-access-restrictions.

Before we proceed with your manuscript, please address the following prompts:

a) If there are ethical or legal restrictions on sharing a de-identified data set, please explain them in detail (e.g., data contain potentially identifying or sensitive patient information, data are owned by a third-party organization, etc.) and who has imposed them (e.g., a Research Ethics Committee or Institutional Review Board, etc.). Please also provide contact information for a data access committee, ethics committee, or other institutional body to which data requests may be sent.

b) If there are no restrictions, please upload the minimal anonymized data set necessary to replicate your study findings to a stable, public repository and provide us with the relevant URLs, DOIs, or accession numbers. Please see http://www.bmj.com/content/340/bmj.c181.long for guidelines on how to de-identify and prepare clinical data for publication. For a list of recommended repositories, please see https://journals.plos.org/plosone/s/recommended-repositories. You also have the option of uploading the data as Supporting Information files, but we would recommend depositing data directly to a data repository if possible.

Please update your Data Availability statement in the submission form accordingly.

7. In the online submission form, you indicated that the datasets used and/or analyzed during the current study are available from the corresponding author on reasonable request.

All PLOS journals now require all data underlying the findings described in their manuscript to be freely available to other researchers, either 1. In a public repository, 2. Within the manuscript itself, or 3. Uploaded as supplementary information.

This policy applies to all data except where public deposition would breach compliance with the protocol approved by your research ethics board. If your data cannot be made publicly available for ethical or legal reasons (e.g., public availability would compromise patient privacy), please explain your reasons on resubmission and your exemption request will be escalated for approval.

8. We note that the grant information you provided in the ‘Funding Information’ and ‘Financial Disclosure’ sections do not match.

When you resubmit, please ensure that you provide the correct grant numbers for the awards you received for your study in the ‘Funding Information’ section.

9. Please include your tables as part of your main manuscript and remove the individual files. Please note that supplementary tables (should remain/ be uploaded) as separate "supporting information" files.

10. We note that there is identifying data in the Supporting Information file <2021_CRT_Px_Model_EPC.pdf>. Due to the inclusion of these potentially identifying data, we have removed this file from your file inventory. Prior to sharing human research participant data, authors should consult with an ethics committee to ensure data are shared in accordance with participant consent and all applicable local laws.

Data sharing should never compromise participant privacy. It is therefore not appropriate to publicly share personally identifiable data on human research participants. The following are examples of data that should not be shared:

-Name, initials, physical address

-Ages more specific than whole numbers

-Internet protocol (IP) address

-Specific dates (birth dates, death dates, examination dates, etc.)

-Contact information such as phone number or email address

-Location data

-ID numbers that seem specific (long numbers, include initials, titled “Hospital ID”) rather than random (small numbers in numerical order)

Data that are not directly identifying may also be inappropriate to share, as in combination they can become identifying. For example, data collected from a small group of participants, vulnerable populations, or private groups should not be shared if they involve indirect identifiers (such as sex, ethnicity, location, etc.) that may risk the identification of study participants.

Additional guidance on preparing raw data for publication can be found in our Data Policy (https://journals.plos.org/plosone/s/data-availability#loc-human-research-participant-data-and-other-sensitive-data) and in the following article: http://www.bmj.com/content/340/bmj.c181.long.

Please remove or anonymize all personal information (<specific identifying information in file to be removed>), ensure that the data shared are in accordance with participant consent, and re-upload a fully anonymized data set. Please note that spreadsheet columns with personal information must be removed and not hidden as all hidden columns will appear in the published file.

11. Please include captions for your Supporting Information files at the end of your manuscript, and update any in-text citations to match accordingly. Please see our Supporting Information guidelines for more information: http://journals.plos.org/plosone/s/supporting-information.

12. If the reviewer comments include a recommendation to cite specific previously published works, please review and evaluate these publications to determine whether they are relevant and should be cited. There is no requirement to cite these works unless the editor has indicated otherwise.

Additional Editor Comments:

1. The authors are invited to submit a revised manuscript addressing all comments from the three reviewers.

2. Data availability: as two reviewers mentioned, the current Data Availability Statement is not sufficient. The authors are advised to carefully review and follow PLOS Data Policy. If there are restrictions of the data, please follow "Acceptable Data Access Restrictions" section.

[Note: HTML markup is below. Please do not edit.]

Reviewers' comments:

Reviewer's Responses to Questions

**Comments to the Author**

1. Is the manuscript technically sound, and do the data support the conclusions?

The manuscript must describe a technically sound piece of scientific research with data that supports the conclusions. Experiments must have been conducted rigorously, with appropriate controls, replication, and sample sizes. The conclusions must be drawn appropriately based on the data presented.

Reviewer #1: No

Reviewer #2: Yes

Reviewer #3: Yes

**********

2. Has the statistical analysis been performed appropriately and rigorously?

Reviewer #1: No

Reviewer #2: Yes

Reviewer #3: Yes

**********

3. Have the authors made all data underlying the findings in their manuscript fully available?

The PLOS Data policy requires authors to make all data underlying the findings described in their manuscript fully available without restriction, with rare exception (please refer to the Data Availability Statement in the manuscript PDF file). The data should be provided as part of the manuscript or its supporting information, or deposited to a public repository. For example, in addition to summary statistics, the data points behind means, medians and variance measures should be available. If there are restrictions on publicly sharing data—e.g. participant privacy or use of data from a third party—those must be specified.

Reviewer #1: Yes

Reviewer #2: No

Reviewer #3: No

**********

4. Is the manuscript presented in an intelligible fashion and written in standard English?

PLOS ONE does not copyedit accepted manuscripts, so the language in submitted articles must be clear, correct, and unambiguous. Any typographical or grammatical errors should be corrected at revision, so please note any specific errors here.

Reviewer #1: Yes

Reviewer #2: Yes

Reviewer #3: Yes

**********

5. Review Comments to the Author

Please use the space provided to explain your answers to the questions above. You may also include additional comments for the author, including concerns about dual publication, research ethics, or publication ethics. (Please upload your review as an attachment if it exceeds 20,000 characters)

Reviewer #1:

The authors aimed to develop a one year survival prediction model for patients with advanced cancer using objective parameters collected in a prospective cohort study. The Cox proportional hazards model was used to identify prognostic factors, and recursive partitioning analysis (RPA) was used to construct the prediction model.

However, there are major methodological concerns that raise questions about the validity of the model building approach.

First, the manuscript restricts the variables entered into the RPA to only those that were statistically significant in the multivariable Cox regression model. This approach is not appropriate for tree based modeling. RPA is designed to detect nonlinear effects, threshold based relationships, and higher order interactions that may not appear significant in a Cox model, which assumes log linearity and does not automatically evaluate interactions. Limiting RPA inputs to only Cox significant variables risks excluding clinically important predictors, introduces bias through data driven preselection, and may reduce the stability and interpretability of the resulting tree.

Second, the same dataset is used for both (1) selecting prognostic factors via Cox regression and (2) developing the RPA prediction model. This constitutes a form of double dipping, in which the data are used twice for variable selection and model construction. Such reuse increases the risk of overfitting, inflates the apparent importance of selected variables, and undermines the validity of the final RPA model. Prognostic factor identification and predictive model development should be conceptually and analytically separated, or at minimum, variable selection should not be based solely on significance testing within the same dataset.

A more robust strategy would be to include all clinically relevant variables (or all variables with acceptable completeness and variability) in the RPA and allow the algorithm to perform its own variable selection. The authors should revise the modeling strategy accordingly and provide justification for the final set of variables included in the tree.

Reviewer #2:

I have carefully reviewed the manuscript entitled “Decision Tree Model to Predict One-Year Survival in Ambulatory Patients with Advanced Cancer”. This manuscript presents a secondary analysis of a South Korean prospective cohort of 200 ambulatory advanced cancer patients with clinician-predicted survival ≤1 year. Using Cox regression and recursive partitioning analysis (RPA), the authors identify prognostic factors and derive a 6-leaf decision tree based on four variables: chemotherapy response, Karnofsky Performance Scale (KPS), CRP/albumin ratio (CAR), and LDH. The topic is clinically relevant, and the decision-tree format is interpretable and potentially useful for palliative care referral decisions.

Strengths:

• Clinically relevant focus on 1-year survival

• Use of objective variables that are commonly available in routine practice (CAR, LDH, KPS, RECIST response)

• The final model is simple and interpretable (tree-based)

Major issues:

• The Data Availability Statement says: “available from corresponding author on reasonable request,” which is generally not sufficient under PLOS data policy unless justified.

• While the decision tree is intuitive, providing more granular performance metrics would make it more useful for clinicians.

o Reporting Sensitivity and Specificity

• Model Validation:

o The authors acknowledge that the study was conducted at a single tertiary center in South Korea. To make the model globally relevant, it should be tested on an independent dataset from a different geographic region or clinical setting. If an external dataset is unavailable, the authors should perform internal validation techniques like k-fold cross-validation or bootstrapping.

Minor issue:

• Some places say CRP <0.08 instead of CAR <0.08 when describing node definitions. Please check carefully for consistent variable naming

• Provide model details for reproducibility

o the R package used for RPA (e.g., rpart, partykit, etc.),

o the selected complexity parameter (cp) value,

o minimum node size, splitting criteria,

o and code or pseudo-code if possible.

• Figure readability

o Figure 1 and Figure 2 appear low resolution and hard to read (node text, axis labels, legend). Please improve figure quality for publication (higher DPI, larger fonts, consistent color/line styles).

Another problem is that the Data availability statement may not be sufficient for PLOS data policy. With minor revisions focused on clarifying model details, adding model validation metrics, and improving figures, the manuscript would be stronger and more reproducible.

Reviewer #3:

This manuscript addresses an important and clinically relevant problem: predicting one-year survival in ambulatory patients with advanced cancer to support timely palliative care referral and shared decision-making. The focus on a one-year horizon fills a notable gap in the prognostication literature, which has largely emphasized short-term outcomes.

Overall, the study is thoughtfully designed and the analyses are competently conducted. The use of a prospective cohort, standardized clinical assessments, and objective laboratory markers strengthens the internal validity of the findings. The combination of Cox proportional hazards modeling with recursive partitioning analysis yields an interpretable and clinically intuitive decision tree based on readily available variables (chemotherapy response, KPS, CAR, and LDH). The identified prognostic factors are well supported by prior literature and biologically plausible.

That said, several limitations should be more clearly emphasized when interpreting the results. First, this is a secondary analysis of a single-center cohort with a relatively modest sample size and heterogeneous cancer types, which may limit generalizability. Second, inclusion based on clinician-predicted survival of ≤1 year introduces potential selection bias, as clinician judgment is subjective and may influence the observed associations. Third, although pruning and cross-validation were performed, the decision tree model remains at risk of overfitting, particularly given the data-driven selection of cut points for continuous variables. The absence of internal optimism correction and external validation further limits conclusions regarding predictive performance and clinical utility.

Besides, the manuscript reports an overall median survival of 7.5 months, while Figure 2 presents median survival times for some terminal nodes exceeding 900 days. Although this may reflect heterogeneity across risk strata, the use of different time units (months vs days) is potentially confusing. The authors should standardize time units across the manuscript and clarify how long survival in low-risk nodes aligns with the inclusion criterion of clinician-predicted survival ≤1 year.

Additionally, the Data Availability Statement indicates that data are available upon reasonable request, which does not meet PLOS ONE data-sharing requirements. If participant privacy restricts full public sharing, the authors should explicitly state these limitations and consider providing a de-identified dataset or depositing the data in a controlled-access repository.

The manuscript is generally well written and clearly organized, with only (1) minor grammatical or stylistic issues that can be addressed during revision (e.g., "LDH levels are also been found..." --> This should be corrected to "LDH levels have also been found to be..."). Figures and tables are clear and appropriately support the text. Some other consistency minor issues need to be fixed as well: (2 Variable Terminology: On page 9, the text describes "Node 2" as having "CRP < 0.08". However, the study focuses on CAR (C-reactive protein/albumin ratio), not raw CRP. This should be corrected for consistency to avoid confusing the two laboratory measures.; (3) Abbreviations: While a list is provided, ensuring that all abbreviations (like CAR and RPA) are consistently defined at their first mention in both the abstract and main text would improve readability.

In summary, this study presents a promising and clinically relevant prognostic model with strong internal coherence. However, conclusions regarding its use in clinical practice should be appropriately tempered until external validation is performed and data-sharing requirements are addressed.

**********

6. PLOS authors have the option to publish the peer review history of their article (what does this mean?). If published, this will include your full peer review and any attached files.

If you choose “no”, your identity will remain anonymous but your review may still be made public.

**Do you want your identity to be public for this peer review?**   For information about this choice, including consent withdrawal, please see our Privacy Policy.

Reviewer #1: No

Reviewer #2: No

Reviewer #3: No

**********

[NOTE: If reviewer comments were submitted as an attachment file, they will be attached to this email and accessible via the submission site. Please log into your account, locate the manuscript record, and check for the action link "View Attachments". If this link does not appear, there are no attachment files.]

To ensure your figures meet our technical requirements, please review our figure guidelines: https://journals.plos.org/plosone/s/figures

You may also use PLOS’s free figure tool, NAAS, to help you prepare publication quality figures: https://journals.plos.org/plosone/s/figures#loc-tools-for-figure-preparation.

NAAS will assess whether your figures meet our technical requirements by comparing each figure against our figure specifications.

# Author comment: Author response to Decision Letter 1

**DOI:** 10.1371/journal.pone.0353195.r002

11 Apr 2026

Thank you for the opportunity to revise our manuscript. We have carefully addressed the comments of the reviewers and listed our point-by-point responses to each below. We express our sincere appreciation for your time and effort and providing your insightful guidance. Our responses are shown in blue.

Response to the Journal Requirements:

Requirement 1:

Please ensure that your manuscript meets PLOS ONE's style requirements, including those for file naming.

Response 1:

Thank you for your comments. We have modified the manuscript accordingly.

Requirement 2:

Please include your tables as part of your main manuscript and remove the individual files. Please note that supplementary tables (should remain/ be uploaded) as separate "supporting information" files.

Response 2:

Thank you for your advice. We’ve included our table(Table 1) as part of our main manuscript and removed the individual files.

Requirement 3:

Please note that PLOS One has specific guidelines on code sharing for submissions in which author-generated code underpins the findings in the manuscript. In these cases, we expect all author-generated code to be made available without restrictions upon publication of the work. Please review our guidelines at https://journals.plos.org/plosone/s/materials-and-software-sharing#loc-sharing-code and ensure that your code is shared in a way that follows best practice and facilitates reproducibility and reuse.

Response 3:

Thank you very much for your advice. We shared our code accordingly and inserted DOI in the manuscript (https://doi.org/10.5281/zenodo.19281393) (p.7, lines 140–141).

Requirement 4:

Your ethics statement should only appear in the Methods section of your manuscript. If your ethics statement is written in any section besides the Methods, please delete it from any other section.

Response 4:

Thank you for your comment. We’ve removed the ethics statement in Declaration section accordingly.

Requirement 5:

We noted in your submission details that a portion of your manuscript may have been presented or published elsewhere. Yes, total dataset was used to publicate a related manuscript. However, the aim was to develop 3-month prediction of survival. This work is different in terms of time-frame. We aimed to develop 1-year survival prediction based on the same dataset. Please clarify whether this [conference proceeding or publication] was peer-reviewed and formally published. If this work was previously peer-reviewed and published, in the cover letter please provide the reason that this work does not constitute dual publication and should be included in the current manuscript.

Response 5:

We appreciate your concern. As you pointed out, we have published the relevant article that aimed to develop 3-month prediction of survival (https://doi.org/10.4143/crt.2021.483). It is peer-reviewed and formally published. We clarified this in current manuscript (p,5, lines 83–87) and the cover letter, too. Though this work used the same dataset, it aimed to develop 1-year survival prediction model. This time-frame is different and predicting model is a brand new one. Therefore, this study had a different research aim, different analysis and results. Thus this does not constitute dual publication.

Requirement 6:

We note that you have indicated that there are restrictions to data sharing for this study. For studies involving human research participant data or other sensitive data, we encourage authors to share de-identified or anonymized data. However, when data cannot be publicly shared for ethical reasons, we allow authors to make their data sets available upon request. Please update your Data Availability statement in the submission form accordingly.

a) If there are ethical or legal restrictions on sharing a de-identified data set, please explain them in detail (e.g., data contain potentially identifying or sensitive patient information, data are owned by a third-party organization, etc.) and who has imposed them (e.g., a Research Ethics Committee or Institutional Review Board, etc.). Please also provide contact information for a data access committee, ethics committee, or other institutional body to which data requests may be sent.

b) If there are no restrictions, please upload the minimal anonymized data set necessary to replicate your study findings to a stable, public repository and provide us with the relevant URLs, DOIs, or accession numbers

Response 6:

Thank you very much for your advice. The local Institutional Review Board (IRB) prohibited to share patient data publicly, because it contains sensitive patient information (survival status, treatment history so on). Original written consent forms from patients didn’t include to share data with others not related to this study directly. Thus it is violation of protection of privacy, according to the local IRB, Seoul National University Bundang Hospital Institutional Review Board. We only can provide the data upon request, and added the contact information of IRB.

The contact information of IRB is as below:

Seoul National University Bundang Hospital Institutional Review Board

Telephone: 082-031-787-8801

Address: 82, Gumi-ro 173 beon-gil, Bundang-gu, Seongnam-si, Gyeonggi-do, 13620, Republic of Korea

Requirement 7:

In the online submission form, you indicated that the datasets used and/or analyzed during the current study are available from the corresponding author on reasonable request. All PLOS journals now require all data underlying the findings described in their manuscript to be freely available to other researchers, either 1. In a public repository, 2. Within the manuscript itself, or 3. Uploaded as supplementary information. This policy applies to all data except where public deposition would breach compliance with the protocol approved by your research ethics board. If your data cannot be made publicly available for ethical or legal reasons (e.g., public availability would compromise patient privacy), please explain your reasons on resubmission and your exemption request will be escalated for approval.

Response 7:

Thank you very much for your advice. As we stated above response 6, public reposition breaches compliance with the protocol approved by our research ethics board. According to the law of privacy protection in Korea, we clarified that patient data would be accessed by only authorized personnel directly related to the study. The law of privacy protection in Korea is quite strict, and it has been reinforced recently. Moreover, the dataset contained sensitive information such as treatment responses, survival status etc. Our research ethics board required that those statements were included in written consent form for patients. Therefore, public availability of this dataset is neither compatible with ethics, nor legal reasons in Korea. We seek your generous understanding about this matter.

Requirement 8:

We note that the grant information you provided in the ‘Funding Information’ and ‘Financial Disclosure’ sections do not match. When you resubmit, please ensure that you provide the correct grant numbers for the awards you received for your study in the ‘Funding Information’ section.

Response 8:

Thank you for pointing this out. We apologize for the mistake and have confirmed the relevant description (p.15, lines 297–298).

Requirement 9:

We note that there is identifying data in the Supporting Information file <2021_CRT_Px_Model_EPC.pdf>. Due to the inclusion of these potentially identifying data, we have removed this file from your file inventory. Prior to sharing human research participant data, authors should consult with an ethics committee to ensure data are shared in accordance with participant consent and all applicable local laws. Data sharing should never compromise participant privacy. It is therefore not appropriate to publicly share personally identifiable data on human research participants. Please remove or anonymize all personal information (<specific identifying information in file to be removed>), ensure that the data shared are in accordance with participant consent, and re-upload a fully anonymized data set. Please note that spreadsheet columns with personal information must be removed and not hidden as all hidden columns will appear in the published file.

Response 9:

Thank you for your opinion. However, the file is an accepted manuscript of a published article, it is not a dataset. It does not contain any personal information. There are only tables and figures published, no columns for identifying data. We changed the file as an officially published article as Supporting Information, to avoid possible misunderstanding.

Requirement 10:

Please include captions for your Supporting Information files at the end of your manuscript, and update any in-text citations to match accordingly.

Response 10:

Thank you for your comment. As we stated above Response 9, Supporting Information file was an accepted manuscript of previously published article using the same dataset. We replaced the file as the officially published file. We included captions for our Supporting Information file at the end of our manuscript. We mentioned the reference as 15 in the reference list.

Requirement 11:

If the reviewer comments include a recommendation to cite specific previously published works, please review and evaluate these publications to determine whether they are relevant and should be cited. There is no requirement to cite these works unless the editor has indicated otherwise.

Response 11:

Thank you for your advice. We understand your requirement. No reviewers recommended more citations.

Response to the reviewer’s comments:

Reviewer 1

Comment 1:

The manuscript restricts the variables entered into the RPA to only those that were statistically significant in the multivariable Cox regression model. This approach is not appropriate for tree based modeling. RPA is designed to detect nonlinear effects, threshold based relationships, and higher order interactions that may not appear significant in a Cox model, which assumes log linearity and does not automatically evaluate interactions. Limiting RPA inputs to only Cox significant variables risks excluding clinically important predictors, introduces bias through data driven preselection, and may reduce the stability and interpretability of the resulting tree.

Response 1:

We appreciate you pointing this out. According to your advice, we applied a new analysis method. First, we put all clinically possible variables – demographic variables, previous treatment effect, symptoms and signs, performance status laboratory tests -- in RPA (p.6, line 126–p.7, line 130). The new results are shown in Abstract (p.2, lines 41–42), Results (p.10, lines 169–179) and Figure 1,2.

Comment 2:

The same dataset is used for both (1) selecting prognostic factors via Cox regression and (2) developing the RPA prediction model. This constitutes a form of double dipping, in which the data are used twice for variable selection and model construction. Such reuse increases the risk of overfitting, inflates the apparent importance of selected variables, and undermines the validity of the final RPA model. Prognostic factor identification and predictive model development should be conceptually and analytically separated, or at minimum, variable selection should not be based solely on significance testing within the same dataset.

Response 2:

Thank you very much for your advice. According to your advice, we decided to change analysis method and used all possible variables in RPA (p.6, line 126–p.7, line 130). The new results are shown in Figure 1 and 2.

Comment 3:

A more robust strategy would be to include all clinically relevant variables (or all variables with acceptable completeness and variability) in the RPA and allow the algorithm to perform its own variable selection. The authors should revise the modeling strategy accordingly and provide justification for the final set of variables included in the tree.

Response 3:

Thank you for your valuable advice. According to your advice, we used all clinically relevant variables (demographic variable, treatment response, symptoms and signs, performance status, body mass index, and laboratory tests) in the RPA (p.6, line 126–p.7, line 130). Those variables were all collected data we had indeed, except tumor type. The new results are shown in Figure 1 and 2.

Reviewer 2

Comment 1:

The Data Availability Statement says: “available from corresponding author on reasonable request,” which is generally not sufficient under PLOS data policy unless justified.

Response 1:

Thank you very much for your advice. The public reposition breaches compliance with the protocol approved by our research ethics board. We clarified that patient data would be accessed by only authorized personnel directly related to the study in the protocol. The law of privacy protection in Korea is quite strict, it has been reinforced recently and it rules the research ethics board. Moreover, the dataset contained sensitive information such as treatment responses, and survival status etc. Our research ethics board required that those statements about privacy protection were included in written consent form for patients. Therefore, public availability of this dataset is neither compatible with ethics, nor legal restrictions in Korea. We seek your generous understanding about this matter.

Comment 2:

While the decision tree is intuitive, providing more granular performance metrics would make it more useful for clinicians. Reporting Sensitivity and Specificity.

Response 2:

We appreciate your advice. For calculating sensitivity and specificity, we need 2*2 table, however the final nodes are 4 nodes. Thus, we need to make the final node to binary variables; i.e. [final node 4] vs. [other nodes], [final node 3 and 4] vs. [node 1 and 2], [final node 2, 3, and 4] vs. [node 1]. Results are as below.

Predictive value Sensitivity Specificity PPV NPV Accuracy

Only 4 0.267 0.971 0.946 0.411 0.510

3 and 4 0.718 0.739 0.839 0.580 0.725

2, 3, and 4 0.962 0.406 0.754 0.848 0.770

We are afraid that these binary classifications do not fit our research aim and analysis model. We do not think these results are appropriate to interpret our analysis. Because we’re interested in each node, grouping nodes seems arbitrary to us. It would be better to show risk of each node calculated in KM curve. Thus, we have decided not to show the results. We hope your generous understanding.

Comment 3:

The authors acknowledge that the study was conducted at a single tertiary center in South Korea. To make the model globally relevant, it should be tested on an independent dataset from a different geographic region or clinical setting. If an external dataset is unavailable, the authors should perform internal validation techniques like k-fold cross-validation or bootstrapping.

Response 3:

Thank you very much for your valuable advice. We performed 10-fold cross-validation (p.7, lines 139–140) and have added the calibration plot as Figure 3.

Comment 4:

Some places say CRP <0.08 instead of CAR <0.08 when describing node definitions. Please check carefully for consistent variable naming.

Response 4:

Thank you for pointing this out. We apologize for the inconsistency. We corrected them accordingly.

Comment 5:

Provide model details for reproducibility;

o the R package used for RPA (e.g., rpart, partykit, etc.),

o the selected complexity parameter (cp) value,

o minimum node size, splitting criteria,

o and code or pseudo-code if possible.

Response 5:

Thank you for your suggestion. We’ve added the details in the code and provided it as DOI (https://doi.org/10.5281/zenodo.19281393) in the manuscript (p.7, lines 140–141).

Comment 6:

Figure 1 and Figure 2 appear low resolution and hard to read (node text, axis labels, legend). Please improve figure quality for publication (higher DPI, larger fonts, consistent color/line styles).

Response 6:

Thank you for your recommendation. We’ve improved the figure quality accordingly and resubmitted those.

Comment 7:

Another problem is that the Data availability statement may not be sufficient for PLOS data policy. With minor rev

Attachment Submitted filename: Response to Reviewers.docx

# Peer review: Decision Letter 1

**DOI:** 10.1371/journal.pone.0353195.r003

**Authors:** Yang Shi, Yang Shi

27 May 2026

PONE-D-25-58729R1

Decision Tree Model to Predict One-Year Survival in Ambulatory Patients with Advanced Cancer

PLOS One

Dear Dr. Suh,

Thank you for submitting your manuscript to PLOS ONE. After careful consideration, we feel that it has been significantly improved but does not yet fully meet PLOS ONE’s publication criteria as it currently stands. Therefore, we invite you to submit a revised version of the manuscript that addresses the points raised during the review process.

Please submit your revised manuscript by Jul 11 2026 11:59PM. If you will need more time than this to complete your revisions, please reply to this message or contact the journal office at plosone@plos.org. When you're ready to submit your revision, log on to https://www.editorialmanager.com/pone/ and select the 'Submissions Needing Revision' folder to locate your manuscript file.

Please include the following items when submitting your revised manuscript:

- A letter that responds to each point raised by the academic editor and reviewer(s). You should upload this letter as a separate file labeled 'Response to Reviewers'.
- A marked-up copy of your manuscript that highlights changes made to the original version. You should upload this as a separate file labeled 'Revised Manuscript with Track Changes'.
- An unmarked version of your revised paper without tracked changes. You should upload this as a separate file labeled 'Manuscript'.

If you would like to make changes to your financial disclosure, please include your updated statement in your cover letter. Guidelines for resubmitting your figure files are available below the reviewer comments at the end of this letter.

If applicable, we recommend that you deposit your laboratory protocols in protocols.io to enhance the reproducibility of your results. Protocols.io assigns your protocol its own identifier (DOI) so that it can be cited independently in the future. For instructions see: https://journals.plos.org/plosone/s/submission-guidelines#loc-laboratory-protocols. Additionally, PLOS ONE offers an option for publishing peer-reviewed Lab Protocol articles, which describe protocols hosted on protocols.io. Read more information on sharing protocols at https://plos.org/protocols?utm_medium=editorial-email&utm_source=authorletters&utm_campaign=protocols.

As the corresponding author, your ORCID iD is verified in the submission system and will appear in the published article. PLOS supports the use of ORCID, and we encourage all coauthors to register for an ORCID iD and use it as well. Please encourage your coauthors to verify their ORCID iD within the submission system before final acceptance, as unverified ORCID iDs will not appear in the published article. *Only*   the individual author can complete the verification step; PLOS staff *cannot*   verify ORCID iDs on behalf of authors.

We look forward to receiving your revised manuscript.

Kind regards,

Yang Shi, PhD

Academic Editor

PLOS One

Journal Requirements:

1. If the reviewer comments include a recommendation to cite specific previously published works, please review and evaluate these publications to determine whether they are relevant and should be cited. There is no requirement to cite these works unless the editor has indicated otherwise.

2. Please review your reference list to ensure that it is complete and correct. If you have cited papers that have been retracted, please include the rationale for doing so in the manuscript text, or remove these references and replace them with relevant current references. Any changes to the reference list should be mentioned in the rebuttal letter that accompanies your revised manuscript. If you need to cite a retracted article, indicate the article’s retracted status in the References list and also include a citation and full reference for the retraction notice.

Additional Editor Comments:

1. Regarding the Data Availability (mentioned by both reviewers), the authors explained in their responses but did not provide a detailed explanation in the Data Availability Statement. The authors need to update the Data Availability Statement following PLOS One policy: https://journals.plos.org/plosone/s/data-availability.

"If there are ethical or legal restrictions on sharing a sensitive data set, authors should provide the following information within their Data Availability Statement upon submission:

- Explain the restrictions in detail (e.g., data contain potentially identifying or sensitive patient information)

- Provide contact information for a data access committee, ethics committee, or other institutional body to which data requests may be sent".

2. The two reviewers mentioned a couple of remaining issues, which I hope the authors can address.

[Note: HTML markup is below. Please do not edit.]

Reviewer's Responses to Questions

**Comments to the Author**

1. If the authors have adequately addressed your comments raised in a previous round of review and you feel that this manuscript is now acceptable for publication, you may indicate that here to bypass the “Comments to the Author” section, enter your conflict of interest statement in the “Confidential to Editor” section, and submit your "Accept" recommendation.

Reviewer #2: (No Response)

Reviewer #3: (No Response)

**********

2. Is the manuscript technically sound, and do the data support the conclusions?

The manuscript must describe a technically sound piece of scientific research with data that supports the conclusions. Experiments must have been conducted rigorously, with appropriate controls, replication, and sample sizes. The conclusions must be drawn appropriately based on the data presented.

Reviewer #2: Yes

Reviewer #3: Partly

**********

3. Has the statistical analysis been performed appropriately and rigorously?

Reviewer #2: Yes

Reviewer #3: Yes

**********

4. Have the authors made all data underlying the findings in their manuscript fully available?

The PLOS Data policy requires authors to make all data underlying the findings described in their manuscript fully available without restriction, with rare exception (please refer to the Data Availability Statement in the manuscript PDF file). The data should be provided as part of the manuscript or its supporting information, or deposited to a public repository. For example, in addition to summary statistics, the data points behind means, medians and variance measures should be available. If there are restrictions on publicly sharing data—e.g. participant privacy or use of data from a third party—those must be specified.

Reviewer #2: No

Reviewer #3: No

**********

5. Is the manuscript presented in an intelligible fashion and written in standard English?

PLOS ONE does not copyedit accepted manuscripts, so the language in submitted articles must be clear, correct, and unambiguous. Any typographical or grammatical errors should be corrected at revision, so please note any specific errors here.

Reviewer #2: Yes

Reviewer #3: Yes

**********

6. Review Comments to the Author

Please use the space provided to explain your answers to the questions above. You may also include additional comments for the author, including concerns about dual publication, research ethics, or publication ethics. (Please upload your review as an attachment if it exceeds 20,000 characters)

Reviewer #2: The authors have addressed most of my comments appropriately, and the manuscript has improved after revision. However, I would appreciate the editor’s guidance regarding two remaining points.

First, although the authors provided a detailed explanation regarding the restrictions on public data sharing due to IRB requirements, patient consent limitations, and Korean privacy regulations, I would like the editor to determine whether the revised Data Availability Statement sufficiently satisfies the journal’s data sharing policy requirements.

Second, while the figures are now generally readable and improved compared with the original submission, the resolution and overall image quality still appear somewhat suboptimal for publication. I would appreciate the editor’s assessment on whether the current figure quality is acceptable for publication standards or if higher-resolution versions should still be requested.

Overall, the manuscript is substantially improved and methodologically sound, and I defer these remaining policy and production-related considerations to the editor’s judgment.

Reviewer #3: Thank you for your thorough revision of the manuscript and for carefully considering the comments from the previous review round. I feel that you have addressed the main issues I raised, and the paper is now close to being suitable for publication, pending minor revision.

In my earlier review I emphasized several limitations that needed clearer discussion: the secondary analysis of a single‑center cohort with a modest sample size and heterogeneous cancer types, the potential selection bias from enrollment based on clinician‑predicted survival ≤1 year, and the risk of overfitting in a data‑driven decision tree model without optimism correction or external validation. In the revision, you have expanded the limitations section to discuss these points more explicitly, and you now present time‑dependent AUC, Brier score, and calibration metrics with internal validation. This is a substantial improvement and better frames the strength and boundaries of the conclusions.

The standardization of time units to days throughout the manuscript and the clarification of why some low‑risk nodes show much longer survival than the overall median directly addresses my previous concern about potential confusion between months and days. Your added explanation about possible reasons for longer survival in the low‑risk group (e.g., treatment responses) is reasonable and appropriately cautious.

I also note improvements in clarity and language. The earlier example I mentioned (“LDH levels are also been found…”) and the inconsistency between CRP and CAR in node descriptions have been corrected, and abbreviations appear to be defined more consistently. The overall structure and flow are clear, and the figures are now more legible.

One point that still merits attention is how you frame the clinical applicability of the model. Even with the improved internal validation, this remains an internally validated model derived from a single institution. I recommend that you slightly soften the language in the Discussion and Conclusions to state explicitly that the decision tree should be viewed as an internally validated prognostic tool that requires external validation in independent cohorts before it can be used routinely in clinical decision‑making.

Finally, I acknowledge your explanation regarding the constraints on data sharing imposed by local privacy law and your IRB. While the ultimate acceptability of this arrangement lies with the journal, it would be helpful if the Data Availability Statement in the manuscript itself clearly and succinctly states: (i) that public deposition of the dataset is not permitted by the IRB; (ii) that any data access must occur under IRB or institutional oversight; and (iii) how interested researchers should initiate such a request. Making this information as specific and transparent as possible will support readers’ understanding of the limitations around data availability.

Overall, this revision addresses my main concerns, and I support publication after these minor textual adjustments.

**********

7. PLOS authors have the option to publish the peer review history of their article (what does this mean?). If published, this will include your full peer review and any attached files.

If you choose “no”, your identity will remain anonymous but your review may still be made public.

**Do you want your identity to be public for this peer review?**   For information about this choice, including consent withdrawal, please see our Privacy Policy.

Reviewer #2: No

Reviewer #3: No

**********

[NOTE: If reviewer comments were submitted as an attachment file, they will be attached to this email and accessible via the submission site. Please log into your account, locate the manuscript record, and check for the action link "View Attachments". If this link does not appear, there are no attachment files.]

To ensure your figures meet our technical requirements, please review our figure guidelines: https://journals.plos.org/plosone/s/figures

You may also use PLOS’s free figure tool, NAAS, to help you prepare publication quality figures: https://journals.plos.org/plosone/s/figures#loc-tools-for-figure-preparation.

NAAS will assess whether your figures meet our technical requirements by comparing each figure against our figure specifications.

# Author comment: Author response to Decision Letter 2

**DOI:** 10.1371/journal.pone.0353195.r004

4 Jun 2026

Thank you for the opportunity to revise our manuscript. We have carefully addressed the comments of the reviewers and listed our point-by-point responses to each below. We express our sincere appreciation for your time and effort and providing your insightful guidance. Our responses are shown in blue.

Response to the Journal Requirements:

Requirement 1:

If the reviewer comments include a recommendation to cite specific previously published works, please review and evaluate these publications to determine whether they are relevant and should be cited. There is no requirement to cite these works unless the editor has indicated otherwise.

Response 1:

Thank you for your comment. There were no recommendations regarding references from the two reviewers.

Requirement 2:

Please review your reference list to ensure that it is complete and correct. If you have cited papers that have been retracted, please include the rationale for doing so in the manuscript text, or remove these references and replace them with relevant current references. Any changes to the reference list should be mentioned in the rebuttal letter that accompanies your revised manuscript. If you need to cite a retracted article, indicate the article’s retracted status in the References list and also include a citation and full reference for the retraction notice.

Response 2:

We thank your advice. We have confirmed reference list accordingly.

Requirement 3:

Regarding the Data Availability (mentioned by both reviewers), the authors explained in their responses but did not provide a detailed explanation in the Data Availability Statement. The authors need to update the Data Availability Statement following PLOS One policy: https://journals.plos.org/plosone/s/data-availability.

"If there are ethical or legal restrictions on sharing a sensitive data set, authors should provide the following information within their Data Availability Statement upon submission:

- Explain the restrictions in detail (e.g., data contain potentially identifying or sensitive patient information)

- Provide contact information for a data access committee, ethics committee, or other institutional body to which data requests may be sent".

Response 3:

We appreciate you pointing this out. We have added the explanation in the section of “Data availability statement” (p.15-16, lines 307–317).

Requirement 4:

The two reviewers mentioned a couple of remaining issues, which I hope the authors can address.

Response 4:

Thank you for your comments. We have addressed the issues accordingly.

Response to the reviewer’s comments:

Reviewer #2

Comment 1:

First, although the authors provided a detailed explanation regarding the restrictions on public data sharing due to IRB requirements, patient consent limitations, and Korean privacy regulations, I would like the editor to determine whether the revised Data Availability Statement sufficiently satisfies the journal’s data sharing policy requirements.

Response 1:

Thank you for your advice. We have revised the “Data availability statement” accordingly (p.15-16, lines 307–317).

Comment 2:

Second, while the figures are now generally readable and improved compared with the original submission, the resolution and overall image quality still appear somewhat suboptimal for publication. I would appreciate the editor’s assessment on whether the current figure quality is acceptable for publication standards or if higher-resolution versions should still be requested.

Response 2:

We thank your valuable advice. We have submitted high-resolution versions of figures as possible.

Reviewer #3

Comment 1:

n my earlier review I emphasized several limitations that needed clearer discussion: the secondary analysis of a single‑center cohort with a modest sample size and heterogeneous cancer types, the potential selection bias from enrollment based on clinician‑predicted survival ≤1 year, and the risk of overfitting in a data‑driven decision tree model without optimism correction or external validation. In the revision, you have expanded the limitations section to discuss these points more explicitly, and you now present time‑dependent AUC, Brier score, and calibration metrics with internal validation. This is a substantial improvement and better frames the strength and boundaries of the conclusions.

The standardization of time units to days throughout the manuscript and the clarification of why some low‑risk nodes show much longer survival than the overall median directly addresses my previous concern about potential confusion between months and days. Your added explanation about possible reasons for longer survival in the low‑risk group (e.g., treatment responses) is reasonable and appropriately cautious.

I also note improvements in clarity and language. The earlier example I mentioned (“LDH levels are also been found…”) and the inconsistency between CRP and CAR in node descriptions have been corrected, and abbreviations appear to be defined more consistently. The overall structure and flow are clear, and the figures are now more legible.

Response 1:

Thank you very much for your comments. We would like to express our gratitude for your previous valuable comments.

Comment 2:

One point that still merits attention is how you frame the clinical applicability of the model. Even with the improved internal validation, this remains an internally validated model derived from a single institution. I recommend that you slightly soften the language in the Discussion and Conclusions to state explicitly that the decision tree should be viewed as an internally validated prognostic tool that requires external validation in independent cohorts before it can be used routinely in clinical decision‑making.

Response 2:

We appreciate your advice. We totally agree with you. We have added the explanation in the section of limitation (p 14 lines 263-264)

Comment 3:

Finally, I acknowledge your explanation regarding the constraints on data sharing imposed by local privacy law and your IRB. While the ultimate acceptability of this arrangement lies with the journal, it would be helpful if the Data Availability Statement in the manuscript itself clearly and succinctly states: (i) that public deposition of the dataset is not permitted by the IRB; (ii) that any data access must occur under IRB or institutional oversight; and (iii) how interested researchers should initiate such a request. Making this information as specific and transparent as possible will support readers’ understanding of the limitations around data availability.

Response 3:

Thank you for your advice. We have revised the “Data availability statement” accordingly (p15-16, lines 307-317).

Closing comments to the editor�

We appreciate the opportunity to revise our manuscript for publication in PLOS One and hope that its revised version meets the journal's standards.

Attachment Submitted filename: Response letter_R2(3).docx

# Peer review: Decision Letter 2

**DOI:** 10.1371/journal.pone.0353195.r005

**Authors:** Yang Shi, Yang Shi, Yang Shi

21 Jun 2026

Decision Tree Model to Predict One-Year Survival in Ambulatory Patients with Advanced Cancer

PONE-D-25-58729R2

Dear Dr. Suh,

We’re pleased to inform you that your manuscript has been judged scientifically suitable for publication and will be formally accepted for publication once it meets all outstanding technical requirements.

Within one week, you’ll receive an e-mail detailing the required amendments. When these have been addressed, you’ll receive a formal acceptance letter and your manuscript will be scheduled for publication.

An invoice will be generated when your article is formally accepted. Please note, if your institution has a publishing partnership with PLOS and your article meets the relevant criteria, all or part of your publication costs will be covered. Please make sure your user information is up-to-date by logging into Editorial Manager at Editorial Manager® and clicking the ‘Update My Information' link at the top of the page. For questions related to billing, please contact billing support.

If your institution or institutions have a press office, please notify them about your upcoming paper to help maximize its impact. If they’ll be preparing press materials, please inform our press team as soon as possible -- no later than 48 hours after receiving the formal acceptance. Your manuscript will remain under strict press embargo until 2 pm Eastern Time on the date of publication. For more information, please contact onepress@plos.org.

Kind regards,

Yang Shi, PhD

Academic Editor

PLOS One

Additional Editor Comments (optional):

Reviewers' comments:

Reviewer's Responses to Questions

**Comments to the Author**

1. If the authors have adequately addressed your comments raised in a previous round of review and you feel that this manuscript is now acceptable for publication, you may indicate that here to bypass the “Comments to the Author” section, enter your conflict of interest statement in the “Confidential to Editor” section, and submit your "Accept" recommendation.

Reviewer #2: All comments have been addressed

Reviewer #3: All comments have been addressed

**********

2. Is the manuscript technically sound, and do the data support the conclusions?

The manuscript must describe a technically sound piece of scientific research with data that supports the conclusions. Experiments must have been conducted rigorously, with appropriate controls, replication, and sample sizes. The conclusions must be drawn appropriately based on the data presented.

Reviewer #2: Yes

Reviewer #3: Yes

**********

3. Has the statistical analysis been performed appropriately and rigorously?

Reviewer #2: Yes

Reviewer #3: Yes

**********

4. Have the authors made all data underlying the findings in their manuscript fully available?

The PLOS Data policy requires authors to make all data underlying the findings described in their manuscript fully available without restriction, with rare exception (please refer to the Data Availability Statement in the manuscript PDF file). The data should be provided as part of the manuscript or its supporting information, or deposited to a public repository. For example, in addition to summary statistics, the data points behind means, medians and variance measures should be available. If there are restrictions on publicly sharing data—e.g. participant privacy or use of data from a third party—those must be specified.

Reviewer #2: Yes

Reviewer #3: Yes

**********

5. Is the manuscript presented in an intelligible fashion and written in standard English?

PLOS ONE does not copyedit accepted manuscripts, so the language in submitted articles must be clear, correct, and unambiguous. Any typographical or grammatical errors should be corrected at revision, so please note any specific errors here.

Reviewer #2: Yes

Reviewer #3: Yes

**********

6. Review Comments to the Author

Please use the space provided to explain your answers to the questions above. You may also include additional comments for the author, including concerns about dual publication, research ethics, or publication ethics. (Please upload your review as an attachment if it exceeds 20,000 characters)

Reviewer #2: I have carefully reviewed the revised manuscript and the authors' responses to my previous comments. The authors have addressed all of my concerns.

I believe the manuscript has been significantly improved and is now suitable for publication. I have no further comments.

Reviewer #3: You have substantially improved the manuscript in response to prior comments. The limitations are now clearly stated, and the statistical analysis—including internal validation with cross‑validation, bootstrap‑corrected time‑dependent AUC, Brier score, and calibration—supports the internal validity of the decision tree. The revised Data Availability Statement is specific and transparent about the IRB‑imposed constraints and the mechanism for requesting data access. Language and figure quality are also improved, and the main results are clearly presented.

Before final acceptance, I suggest only one minor addition: please add 1–2 sentences in the Methods or Discussion briefly justifying the choice of a simple decision tree (recursive partitioning) over alternative approaches (e.g., Cox model, penalized regression, or ensemble tree methods), highlighting the trade‑off between interpretability and potential gains in predictive performance. This clarification will help readers understand the modeling rationale, but no further analyses are required.

**********

7. PLOS authors have the option to publish the peer review history of their article (what does this mean?). If published, this will include your full peer review and any attached files.

If you choose “no”, your identity will remain anonymous but your review may still be made public.

**Do you want your identity to be public for this peer review?**   For information about this choice, including consent withdrawal, please see our Privacy Policy.

Reviewer #2: No

Reviewer #3: No

**********

# Editor report: Acceptance letter

**DOI:** 10.1371/journal.pone.0353195.r006

**Authors:** Yang Shi, Yang Shi, Yang Shi

PONE-D-25-58729R2

PLOS One

Dear Dr. Suh,

I'm pleased to inform you that your manuscript has been deemed suitable for publication in PLOS One. Congratulations! Your manuscript is now being handed over to our production team.

At this stage, our production department will prepare your paper for publication. This includes ensuring the following:

* All references, tables, and figures are properly cited

* All relevant supporting information is included in the manuscript submission,

* There are no issues that prevent the paper from being properly typeset

You will receive further instructions from the production team, including instructions on how to review your proof when it is ready. Please keep in mind that we are working through a large volume of accepted articles, so please give us a few days to review your paper and let you know the next and final steps.

Lastly, if your institution or institutions have a press office, please let them know about your upcoming paper now to help maximize its impact. If they'll be preparing press materials, please inform our press team within the next 48 hours. Your manuscript will remain under strict press embargo until 2 pm Eastern Time on the date of publication. For more information, please contact onepress@plos.org.

You will receive an invoice from PLOS for your publication fee after your manuscript has reached the completed accept phase. If you receive an email requesting payment before acceptance or for any other service, this may be a phishing scheme. Learn how to identify phishing emails and protect your accounts at https://explore.plos.org/phishing.

If we can help with anything else, please email us at customercare@plos.org.

Thank you for submitting your work to PLOS ONE and supporting open access.

Kind regards,

PLOS ONE Editorial Office Staff

on behalf of

Dr. Yang Shi

Academic Editor

PLOS One
